# The Human TREX-2 Complex Interacts with Subunits of the ORC Complex

**DOI:** 10.1134/S1607672923700552

**Published:** 2023-12-08

**Authors:** M. M. Kurshakova, S. G. Georgieva, D. V. Kopytova

**Affiliations:** Institute of Molecular Biology, Russian Academy of Sciences, Moscow, Russia

**Keywords:** TREX-2, GANP, PCID2, Xmas-2, ORC, Orc3, Orc4, mRNA export

## Abstract

The TREX-2 protein complex is the key complex involved in the export of mRNA from the nucleus to the cytoplasm through the nuclear pores. Previously, a joint protein complex of TREX-2 with ORC was isolated in *D. melanogaster*. It was shown that the interaction of TREX-2 with ORC is necessary for efficient mRNA export from the nucleus to the cytoplasm. In this work, we showed that the TREX-2-ORC joint complex is also formed in human cells.

Gene expression includes the stages of mRNA synthesis, formation of the mature mRNP, and export of mRNA from the nucleus to the cytoplasm through the nuclear pores. During the formation of the mRNP various proteins regulating processing, mRNA interaction with nuclear pores, and mRNA export are incorporated into it. Previously, in *D. melanogaster*, we described the TREX-2 complex as a protein component of mRNP [1]. It was shown that TREX-2 is associated with the nuclear pore and is necessary for the export of mRNA from the nucleus to the cytoplasm. Complexes homologous to TREX-2 were characterized in many eukaryotes, including yeast and humans, and TREX-2 was shown to play the key role in mRNA export in various organisms [2–4]. The TREX-2 complex of *D. melanogaster* consists of the Xmas-2, PCID2, ENY2, and Sem1p proteins. The platform for the complex assembly is the Xmas-2 protein, with which other proteins interact. On the basis of the data on the interaction of proteins in TREX-2 that were obtained for the Sac3 protein, yeast Xmas-2 homologue, and comparison of protein sequences the domain structure of Xmas-2 was deduced. It was assumed that Xmas-2 has an RNA-binding domain (RRM) at the N terminus. Further, in the 3'-end region of the Sac3-GANP domain, there is a domain with which PCID2 and mRNA bind. Closer to the C terminus of the protein, there is a CID domain, with which ENY2, associated with the nuclear pore, binds [5–8]. Our team isolated a joint complex of TREX-2 with proteins of the ORC complex (Origin Recognition Complex) and demonstrated the role of this interaction in the export of mRNA from the nucleus [9]. The ORC complex was first described in budding yeast as a complex involved in replication initiation [10]. Later, homologous complexes were discovered in other organisms. The ORC complex is involved in the recruitment of the Mcm2-7 complex to replication origins with the participation of the Cdc6 and Cdt1 factors [11]. However, in higher eukaryotes, the ORC complex and its individual subunits exhibit additional properties not associated with replication [12]. In particular, it was shown that ORC proteins interact with various RNAs [13]. We showed that ORC proteins interact with the mRNP, and this interaction is mediated by the TREX-2 complex [9]. ORC subunits were shown to interact with the NXF1 export receptor and are required for the binding of NXF1 to the mRNP. Knockdown of ORC components impairs mRNA export, thus demonstrating an important role of ORC in the mRNA export pathway. However, it remains unclear whether or not this mechanism is universal. To answer this question, we studied possible interaction between the TREX-2 complex subunits and the ORC complex subunits in human cell culture.

In humans, the TREX-2 complex consists of the GANP protein (Xmas-2 homologue), the PCID2 protein, two copies of the ENY2 protein, the DSS1 protein (Sem1p homologue), and centrins CETN2/CETN3 (yeast Cdc31 protein homologues) [4]. The ORC complex is evolutionarily conserved and consists of six subunits—Orc1, Orc2, Orc3, Orc4, Orc5, and Orc6 [14]. Although proteins Orc2–Orc5 coprecipitate with each other in human cell lysates under mild extraction conditions, joint complex of subunits is difficult to isolate. Extraction under harsher conditions led to coprecipitation of several Orc proteins with stoichiometric amounts of other unidentified proteins, but not with one of the known ORC subunits [15].

Previously, Orc1, Orc3, Orc4, Orc5, and Orc6 subunits were found in the purified joint TREX-2–ORC complex of *D. melanogaster*. The direct interaction of each of the identified ORC proteins with TREX-2 components (Xmas-2, PCID2, and ENY2) was investigated [9]. We showed that, of all ORC proteins, the Orc3 subunit was the most strongly associated with the TREX-2 complex and the mRNP particle, binding together with ENY2 to the C terminus of Xmas-2 [9, 16, 17]. Orc4 binds most efficiently to PCID2 [9]. On the basis of these data, to investigate the existence of a protein complex homologous to the TREX-2–ORC complex in humans, we decided to obtain antibodies to the GANP and PCID2 subunits of human TREX-2 and to the Orc3 and Orc4 subunits of human ORC. The domain structure of the proteins was analyzed and amino acid sequence regions for the synthesis of antigens were selected (Fig. 1a). For GANP, the 1050–1247 aa fragment, containing the CID domain sequence, was selected; for PCID2, the 11–399 aa fragment, almost completely identical to the full-length protein sequence; for Orc3 and Orc4, 534–711 aa and 236–436 aa fragments, respectively, overlapping the C-terminal domains of the proteins that contain the DNA-binding WH domains [11]. Genetic constructs encoding the fragments of amino acid sequences of the proteins in frame with the His-tag were created. Then, the resulting constructs were used to express the recombinant proteins in *E. coli*. The proteins were purified from bacterial lysates using Ni-NTA agarose. Next, rabbits were immunized with the corresponding purified proteins. Polyclonal antibodies to human proteins GANP, PCID2, Orc3, and Orc4 were affinity purified from the blood serum of the immunized animals. The specificity of the antibodies was tested in Western blot analysis of total lysates of human HEK293T cells (Fig. 1b). The antibodies to GANP recognized the full-length protein and the proteolytic fragments of approximately 115 and 80 kDa. It was previously shown that, in HEK293 cells, GANP can undergo proteolysis to form a C-terminal fragment about 110–115 kDa; the protein is cleaved in the region 1024–1029 aa [18]. Since our antibodies were generated to the GANP region 1050–1247 aa, they can recognize both the full-length protein and C-terminal proteolytic fragments. Earlier, we showed that *D. melanogaster* Xmas-2 is also cut into the N-terminal and C-terminal fragments [19]. The C-terminal fragment of Xmas-2 was detected as a 100-kDa band, as well as an 80-kDa band, which apparently was a product of further protein degradation. Thus, the generated antibodies to GANP specifically recognized GANP and its C-terminal proteolytic fragments. The antibodies to Orc3 specifically recognized a protein of approximately 80 kDa, which is close to it’s predicted mass. The antibodies to Orc4 specifically recognized a protein of about 45 kDa, which is close to the calculated mass. The antibodies to PCID2 recognized both the major form of the protein (~41 kDa) and two minor modified forms of approximately 46 and 67 kDa. Previously, we showed that, in *D. melanogaster* cells, PCID2 is present in three forms of about 42, 47, and 52 kDa. The 42-kDa form corresponds to the predicted mass of PCID2, and the 47- and 52-kDa forms are ubiquitinylated modifications of PCID2 [20]. It can be assumed that PCID2 is also present in human cells in the major and modified forms.

**Fig. 1.**
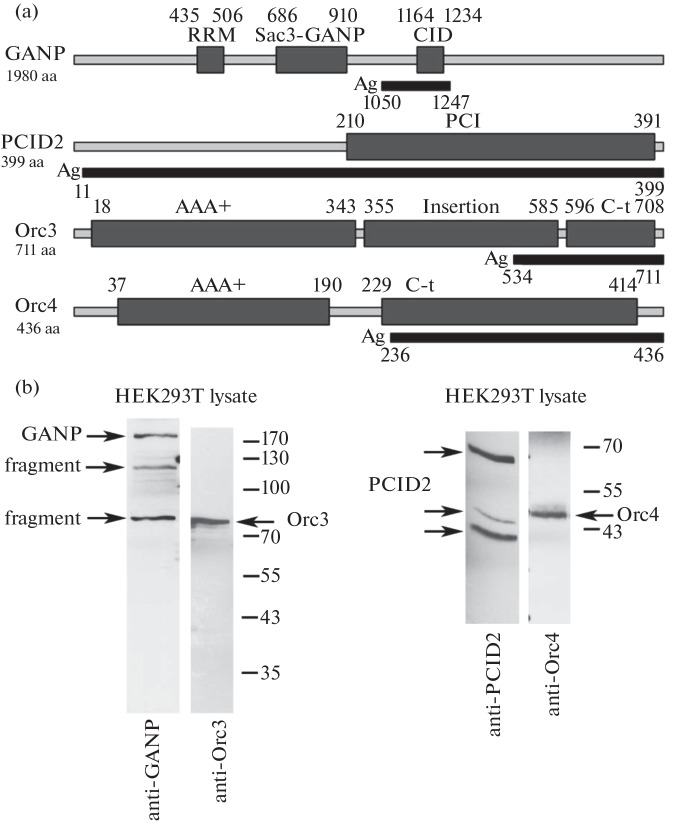
Production of the polyclonal antibodies to the subunits of TREX-2 and ORC complexes. (a) Scheme of the amino acid sequences of GANP, PCID2, Orc3, and Orc4 proteins. The main domains of the proteins are indicated. The sequences used for antibody production (Ag) are indicated in black rectangles. (b) Analysis of the antibody specificity. Western blot analysis of HEK293T cell lysate using affinity-purified antibodies to GANP, PCID2, Orc3, and Orc4. Arrows indicate the protein forms corresponding to their molecular mass, as well as proteolytic forms of GANP (fragment) and the modified forms of PCID2.

Next, to test possible interaction between the TREX-2 and ORC complexes in human cells, we carried out immunoprecipitation experiments of the TREX-2 subunits GANP and PCID2 with the ORC subunits Orc3 and Orc4 from total lysates of HEK293T cells (Fig. 2). To exclude indirect interaction of TREX-2 and ORC subunits through binding to DNA or RNA, immunoprecipitaion reactions were performed in lysates treated with DNase I and RNase A. Cell lysates were isolated with the addition of phosphatase inhibitors (Sigma) to maintain the phosphorylated state of proteins. Phosphorylation of proteins is often required for the formation of protein complexes and maintaining their structure.

**Fig. 2.**
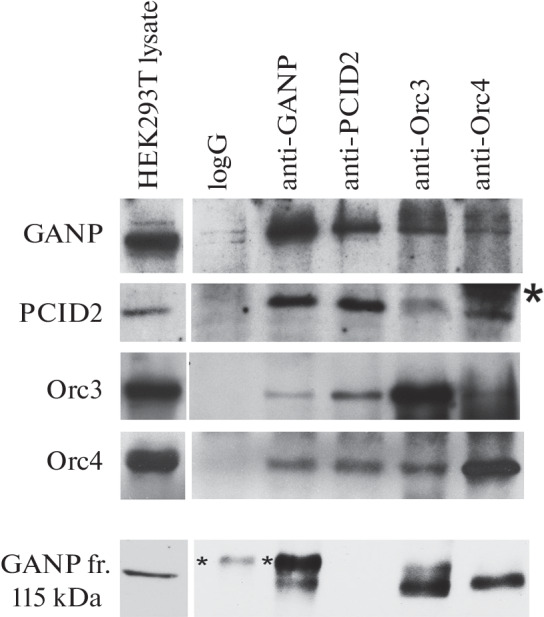
Analysis of the interaction of TREX-2 and ORC components. Western-blot analysis of the immunoprecipitation of full-length proteins and the C-terminal GANP fragment (lower panel) from HEK293T cell lysates using antibodies to TREX-2 subunits, GANP and PCID2 proteins, and ORC subunits, Orc3 and Orc4 proteins. The detected proteins are indicated. The bands corresponding to the staining of antibodies chains are marked with asterisks.

All antibodies effectively functioned in immunocoprecipitation reactions, precipitating the proteins to which they were generated. Under our conditions, the antibodies to PCID2 precipitated predominantly the major low-molecular weight form of PCID2 of approximately 41 kDa and poorly precipitated the other two forms of PCID2. The antibodies to GANP precipitated the major form of the protein and also a 115 kD GANP fragment (Fig. 2, bottom panel). The antibodies to GANP coprecipitated the major form of PCID2, and the antibodies to PCID2 coprecipitated the full-length GANP. In *D. melanogaster*, the higher-molecular-weight forms of PCID2 are modifications of the major form and, unlike the major form, do not bind to Xmas-2 [20]. GANP also was not associated with the high-molecular weight forms of PCID2. Since GANP and PCID2 bind each other in the TREX-2 complex, both GANP and PCID2 antibodies coprecipitate TREX-2. The antibodies to GANP and PCID2 effectively coprecipitated both Orc3 and Orc4. The antibodies to Orc3 and Orc4 poorly coprecipitated each other, which is consistent with the published data that ORC components in many tissues have different levels of expression and are difficult to isolate together in one complex [15]. The antibodies to Orc3 and Orc4 coprecipitated the full-length GANP protein. In addition, the antibodies to Orc3 and Orc4 coprecipitated the low-molecular weight form of PCID2. Thus, in human HEK293T cells, some part of the ORC proteins are associated with the TREX-2 complexes.

It is likely that, in the joint complex, Orc3 can directly interact with both GANP and PCID2, since antibodies to both proteins precipitate Orc3 effectively enough. Interestingly, PCID2 coprecipitates quite efficiently with Orc4, which may indicate their direct interaction in the complex. Our results suggest that multiple interactions exist within the joint complex between ORC and TREX-2 subunits. The study of direct interactions of subunits within the joint TREX-2–ORC complex of *D. melanogaster* showed that Orc3 most effectively binds to the Xmas-2 and ENY2 subunits of TREX-2 [9]. Orc3, in complex with ENY2, associates with the C-terminal region of Xmas-2, which contains the CID domain [16, 17]. Orc4 binds most efficiently to PCID2, and Orc3 also binds to PCID2 [9]. Our data suggest that, in human cells, the joint complex of TREX-2 with ORC proteins may have a similar structure to the TREX-2–ORC complex of *D. melanogaster*.

Interestingly, when HEK293T cell lysates were obtained in the presence of phosphatase inhibitors, the antibodies to Orc3 and Orc4, as well as the antibodies to GANP, coprecipitated the 115 kDa C-terminal proteolytic GANP fragment (Fig. 2, bottom panel). It is worth noting that, in immunocoprecipitations from *D. melanogaster* S2 cell lysates obtained in the presence of phosphatase inhibitors, antibodies to Orc3 can also coprecipitate the C-terminal proteolytic fragment of Xmas-2. One can propose that, under certain conditions, ORC proteins are able to associate not only with the full-length GANP, but also with its C-terminal proteolytic fragment.

The studied components of TREX-2 and ORC were also coprecipitated from human A375 cell lysates in immunocoprecipitation reactions under similar conditions. Thus, in human cells of various lines, interaction between the TREX-2 complex and ORC complex subunits was discovered. We suggest that a joint complex of TREX-2 with ORC proteins, similar to TREX-2-ORC complex of D. melanogaster, can form in human cells. Further research is required to determine its structure and functions.
